# Preclinical Bioavailability, Tissue Distribution, and Protein Binding Studies of Erinacine A, a Bioactive Compound from *Hericium erinaceus* Mycelia Using Validated LC-MS/MS Method

**DOI:** 10.3390/molecules26154510

**Published:** 2021-07-27

**Authors:** Pei-Ching Tsai, Yi-Kai Wu, Jun-Hao Hu, I-Chen Li, Ting-Wei Lin, Chin-Chu Chen, Chia-Feng Kuo

**Affiliations:** 1 Department of Food Science, Nutrition, and Nutraceutical Biotechnology, Shih Chien University, Taipei 10462, Taiwan; neoneo@g2.usc.edu.tw (P.-C.T.); pssn4500@gmail.com (Y.-K.W.); Polo5566789@gmail.com (J.-H.H.); gkbioeng@grapeking.com.tw (C.-C.C.); 2 Biotech Research Institute, Grape King Bio Ltd., Taoyuan 32542, Taiwan; Ichen.li@grapeking.com.tw (I.-C.L.); tingwei.lin@grapeking.com.tw (T.-W.L.)

**Keywords:** *Hericium erinaceus* mycelia, erinacine A, bioavailability, tissue distribution, excretion

## Abstract

Erinacine A, derived from the mycelia of *Hericium erinaceus*, has attracted much attention due to its neuroprotective properties. However, very few studies have been conducted on the bioavailability, tissue distribution, and protein binding of erinacine A. This study aimed to investigate the bioavailability, tissue distribution, and protein binding of erinacine A in Sprague-Dawley rats. After oral administration (po) and intravenous administration (iv) of 2.381 g/kg BW of the *H. erinaceus* mycelia extract (equivalent to 50 mg/kg BW of erinacine A) and 5 mg/kg BW of erinacine A, respectively, the absolute bioavailability of erinacine A was estimated as 24.39%. Erinacine A was detected in brain at 1 h after oral dosing and reached the peak at 8 h. Protein binding assay showed unbound erinacine A fractions in brain to blood ratio is close to unity, supporting passive diffusion as the dominating transport. Feces was the major route for the elimination of erinacine A. This study is the first to show that erinacine A can penetrate the blood-brain barrier of rats by the means of passive diffusion and thus support the development of *H. erinaceus* mycelia for the improvement of neurohealth.

## 1. Introduction

*Hericium erinaceus* is a culinary-medicinal mushroom with a long history of usage in Eastern Asia [1]. This mushroom has been widely reported to possess wound healing [2], anti-hyperlipidemic [3], anti-hyperglycemic [3], ischemia injury-preventive [4], anti-cancer [5], anti-bacterial [6], immuno-modulating [7], antioxidant [8], anti-osteoporotic [8], anti-neurodegenerative [9], anti-depression [10], and Alzheimer’s disease-ameliorating [11] activities. Due to these potential health benefits, *H. erinaceus* has attracted attention for the research and development of functional foods.

Erinacines are the major compounds derived from the mycelia of *H. erinaceus*. Among the 15 erinacines identified (erinacines A–K and P–S), erinacine A has attracted most attention due to its neuroprotective properties [9]. Erinacine A from the mycelia of *H. erinaceus*. not only stimulated nerve growth factor (NGF) in vitro [12] but also increased NGF content in hippocampus and locus coeruleus of rats after administration (8 mg/kg BW) for 5 weeks from birth [13]. Meta-analysis has shown that NGF concentrations are significantly lower in patients with major depressive disorder than in healthy subjects [14], so erinacine A and *H. erinaceus*. mycelia enriched with erinacine A (>5 mg/g) are hypothesized to alleviate depression. Oral administration of erinacine A-enriched *H. erinaceus* mycelia to stressed mice (200 mg/kg/day) significantly reduced immobility time in the tail suspension test (TST) and forced swimming test (FST), and number of entries in open arm (PTOA) [10].

In a rat model of transient focal cerebral ischemia, intraperitoneal administration of erinacine A (1 mg/kg) during the 5 days before the onset of ischemia significantly reduced the levels of acute inflammatory cytokines and ischemia-injury-induced neuronal cell death [4]. Inhibition on amyloid-β plaque formation has become a therapeutic target for primary prevention of Alzheimer’s disease [15]. In 5-month-old APPswe/PS1dE9 transgenic mice, treatment with erinacine A-enriched *H. erinaceus* mycelia and its ethanol extract for 30 days (both 300 mg/kg/day) attenuated cerebral Aβ plaque burden [11].

In addition to the neurohealth property, treatments with erinacine A inhibited the proliferation of DLD-1 colorectal adenocarcinoma cells in vitro and the growth of DLD-1 xenografts in nude mice [16,17]. Moreover, treatment with erinacine A-enriched *H. erinaceus* mycelia promoted longevity in senescence-accelerated P8 (SAMP8) mice [18].

Although it is difficult to extrapolate in-vitro and in-vivo studies to clinical application, preclinical studies have shown that intake of erinacine A-enriched *H. erinaceus* mycelia can have improvement in neurodegenerative diseases. To date, animal studies have shown no adverse effects of erinacine A-enriched *H. erinaceus* mycelia when administered up to 5 g/kg and 3 g/kg for acute exposure and 28-d feeding, respectively [19,20,21]. However, no studies have ever shown erinacine A in mycelia could be absorbed into circulation and localized in the brain. From our previous study, another compound isolated from *H. erinaceus* mycelia erinacine S, was shown to penetrate the blood-brain barrier of rats [22]. Despite both erinacine S and erinacine A are isolated from the same source, they have different physicochemical and biological properties [23]. In fact, prior reports showed that erinacine A but not erinacine S could reduce the level of insoluble β amyloid and C-terminal fragment of amyloid precursor protein [24]. Hence, it is worth investigating the pharmacokinetic disposition of erinacine A. Therefore, this is the first study to explore the preclinical pharmacokinetics and protein binding of erinacine A after oral administration of *H. erinaceus* mycelia. It was expected that the results of this study will support the development of erinacine A-rich *H. erinaceus* mycelia for the improvement of neurohealth.

## 2. Materials and Methods

### 2.1. Preparation of H. erinaceus Mycelia Extract and Erinacine A

*H. erinaceus* purchased from the Bioresources Collection and Research Center in Food Industry Research and Development Institute (BCRC 35669; Hsinchu, Taiwan) was cultured on potato dextrose agar at 26 °C for 15 days. For liquid fermentation, the fungal mycelia were cut from the agar and grown in 2-L flasks containing 1.3 L of synthetic medium (4.5% glucose, 0.5% soybean powder, 0.25% yeast extract, 0.25% peptone, and 0.05% MgSO_4_, adjusted to pH 4.5) on a rotary shaker incubator (Model S103, Firstek Scientific, Taipei, Taiwan) at 120 rev/min at 25 °C for 5 days. Then the flasks were scaled up to 500-L fermenters for 5 days before further scaled up to 20-ton fermenters for another 12 days (Grape King Bio, Taoyuan, Taiwan). After cultivation, the mycelia were harvested, lyophilized, grounded, and then extracted by mixing and sonicating with 95% ethanol, followed by filtration and concentration. Erinacine A was extracted and determined according to a previous report [12]. Briefly, the extract was fractionated by solvent partition between H_2_O and ethyl acetate (EtOAc). The ethyl acetate fraction was applied onto a silica gel column (70–230 mesh, 70 × 10 cm) using a gradient system of *n*-hexane/EtOAc to provide fractions (Nacalai USA, Inc., San Diego, CA, USA). Erinacine A from the fractions was isolated according to the methods previously reported [19].

### 2.2. Bioanalysis of Erinacine A

Analysis of erinacine A concentration in samples was conducted by Agilent 1100 series HPLC system (Agilent, Waldbronn, Germany) equipped with a G1376A capillary pump and a G1313A autosampler. Chromatographic separation was carried out in an Agilent Eclipse XDB-C18 column (3.5 µm, 4.6 × 100 mm) at 25 °C. The mobile phase consists of water (A) and acetonitrile (B) with gradient elution program as follows: 0 min, 70% B; 0–5 min, 70–100% B; 5–8 min, 100% B; 8–8.1 min, 100–70% B; 8.1–11 min, 70% B. The flow rate was 0.35 mL/min and injection volume was 10 μL. Mass spectrometric detection was conducted on an API 3000 triple quadrupole instrument (Applied Biosystems, Vaughan, ON, Canada) in multiple reaction monitoring (MRM) mode with parameters as follows: nebulizer gas, 10 psi; collision gas, 2 psi; curtain gas, 7 psi; source voltage, 4.5 kV; source temperature, 275 °C. A turbo ionspray electrospray ionization (ESI) interface in positive ionization mode was used (Applied Biosystem, Foster City, CA, USA). The specific precursor to product ion transitions is [M+H] 433 → 301 for erinacine A and 197 → 169 for 2,4,5-TMBA (internal standard). Data analysis was performed with Analyst 1.4.2 software (Applied Biosystems, Concord, ON, Canada) [22].

### 2.3. Method Validation

LC/MS/MS method was validated according to the guidelines set by the European Medicines Agency [25]. Intra- and inter-day variations were measured by analyzing erinacine A at 7 concentration levels (5, 10, 20, 50, 100, 200, and 500 ng/mL) in six replicate samples on three consecutive validation days. The methods are acceptable when the precision and accuracy of intra-day and inter-day are within ±15% [26]. Precision (measure of the random error) and accuracy (measure of systematic error). were expressed as percentage coefficient of variation (% CV) and percentage bias (% bias), respectively. Percentage coefficient of variation is calculated as (standard deviation/mean) × 100 while percentage bias is calculated as [(measured concentration − theoretical concentration)/theoretical concentration] × 100 [26].

Recoveries were assessed by comparing the chromatograms of samples which were spiked with erinacine A and internal standard 2,4,5-TMBA (Sigma-Aldrich, St. Louis, MO, USA) before extraction to that of samples which were spiked after extraction. Briefly, 40 μL of erinacine A stock solution at different concentrations were added to 160 μL of plasma or tissue homogenates to make working solution. Two hundred μL of internal standard 2,4,5-TMBA (final concentration 200 ng/mL) and 800 μL ethyl acetate was added to the solution followed by vortexing for 1 min and centrifuging at 9615× *g* for 10 min. The supernatant was evaporated to dryness and the residue was reconstituted with isovolumetric acetonitrile for LC-MS/MS analysis. Extraction recovery (%) was calculated as: (measured concentration/theoretical concentration) × 100% [22].

### 2.4. Pharmacokinetic Study

Twelve 8-week-old male Sprague-Dawley rats were purchased from BioLASCO Co (Taipei, Taiwan) and maintained at 22 ± 2 °C with a 12:12 h light–dark cycle. Water and chow diet (MF 18 Rodent diet, Oriental Yeast Co., Tokyo, Japan) were given ad libitum. When animals weighted 280–300 g, they were randomly assigned into two groups (n = 6) according to a computer-generated randomization list: oral administrated with *H. erinaceus* mycelia extract (2.381 g/kg body weight; equivalent to 50 mg/kg BW of erinacine A) or intravenous administration with erinacine A (5 mg/kg BW). Mycelia extract was dissolved in distilled H_2_O containing 10% DMSO and erinacine A was dissolved in DMSO. Based on previous studies, DMSO has low acute toxicity by oral and intravenous routes [27].

Before intravenous administration, animals were anesthetized with Avertin (2,2,2-Tribromoethanol, 0.071 M). Blood was collected into heparinized microtubes at scheduled time points and centrifuged at 2404× *g* for 10 min. The plasma (100 μL) was then mixed with equal volume of internal standard 2,4,5-TMBA (final concentration 25 ng/mL) and 300 μL of ethyl acetate. The mixture was vortexed and centrifuged at 13,845× *g* for 3 min. The supernatant was removed and the residue was reconstituted with isovolumetric acetonitrile for LC-MS/MS analysis [22]. All animal procedures were approved by the Animal Care and Use Committee of Shih Chien University (IACUC#10405).

### 2.5. Distribution Study

For the tissue distribution study, animals received a single oral administration of *H. erinaceus* mycelia extract at 2.381 g/kg (equivalent to 50 mg/kg BW of erinacine A) and sacrificed at 0, 0.5, 1, 2, 4, 8, 12, or 24 h after administration (n = 3 at each time point). Brain, heart, lung, liver, kidney, and gastrointestinal tract (chyme was removed before the wash) were collected and washed by 0.9% NaCl before homogenization. The homogenates (100 μL) were then mixed with equal volume of internal standard 2,4,5-TMBA (final concentration 25 ng/mL) and 300 μL of ethyl acetate. The mixture was vortexed and centrifuged at 13,845× *g* for 3 min. The supernatant was removed and the residue was reconstituted with isovolumetric acetonitrile for LC-MS/MS analysis.

### 2.6. Excretion Study

For the excretion study, feces and urine were collected at 0–4, 4–8, 8–12, and 12–24 h (n = 6) after animals received a single oral administration of *H. erinaceus* mycelia extract at 2.381 g/kg (equivalent to 50 mg/kg BW of erinacine A). Feces were freeze-dried and grinded before mixing with equal volume of internal standard 2,4,5-TMBA (final concentration 25 ng/mL) and 300 μL of ethyl acetate. Urines were mixed with equal volume of internal standard 2,4,5-TMBA (final concentration 25 ng/mL) and 300 μL of ethyl acetate. The mixture was vortexed and centrifuged at 13,845× *g* for 3 min. The supernatant was removed and the residue was reconstituted with isovolumetric acetonitrile for LC-MS/MS analysis.

### 2.7. Protein Binding Study

To confirm that erinacine A does not degrade over the time-course of protein binding study, the stability of erinacine A in plasma and tissues from wild Sprague-Dawley rats at 37 °C for 4 h was first evaluated to show that no degradation was observed (data not shown). Then, the protein binding of erinacine A in the plasma and diluted (4×) tissue homogenates (brain, heart, lung, liver, kidney, stomach, large intestine, and small intestine) was determined by rapid equilibrium dialysis devices with 8000 Da molecular weight cut-off (#89809, Thermo Fisher Scientific, Waltham, MA, USA) according to manufacturer’s guidelines. In brief, the stock solution of erinacine A prepared in methanol was spiked into blank plasma and tissues to yield a final concentration of 200 ng/mL. Two hundred microliter aliquot of spiked samples were then dialysed against 350 µL aliquot of isotonic phosphate buffered saline at 37 °C on an orbital shaker at 250 rpm for 4 h. The resulting sample and buffer dialysates were removed from the chambers, matched with an equal volume of opposite blank matrix, and analyzed by LC-MS/MS. The protein binding of erinacine A is determined by the following Equations (1) and (2):

% Free = (concentration at buffer chamber/concentration at sample chamber) × 100%(1)

% Bound = 100% − % Free(2)

### 2.8. Data Analysis

All experimental data were presented as mean ± standard deviation (mean ± SD). Maximal plasma concentration (C_max_), and time taken to achieve maximal plasma concentration (T_max_), half-life (T_1/2_), and area under the plasma concentration − time curve (AUC) were assessed on each animal using the software of WinNonlin (Pharsight Corp., Mountain View, CA, USA) by the non-compartmental model. Applying the pharmacokinetic data of oral administration (po) and intravenous administration (iv), the absolute bioavailability of erinacine A was calculated as [(AUC_po_ × Dose_iv_)/(AUC_iv_ × Dose_po_)] × 100%.

## 3. Results

### 3.1. Method Validation

**Figure 1:**
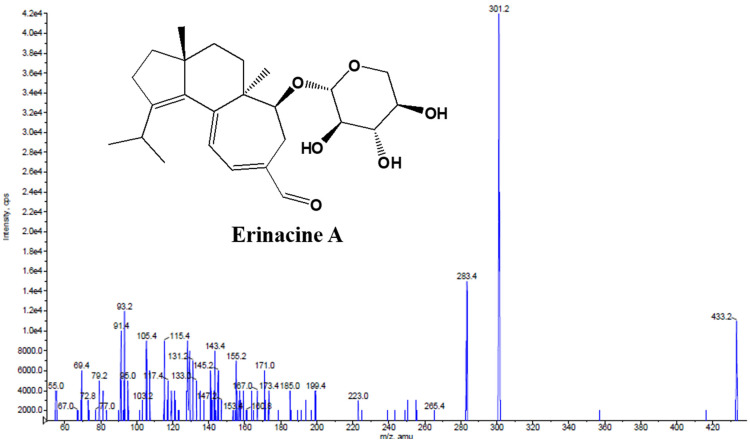
Product ion spectrum of erinacine A.

**Figure 2:**
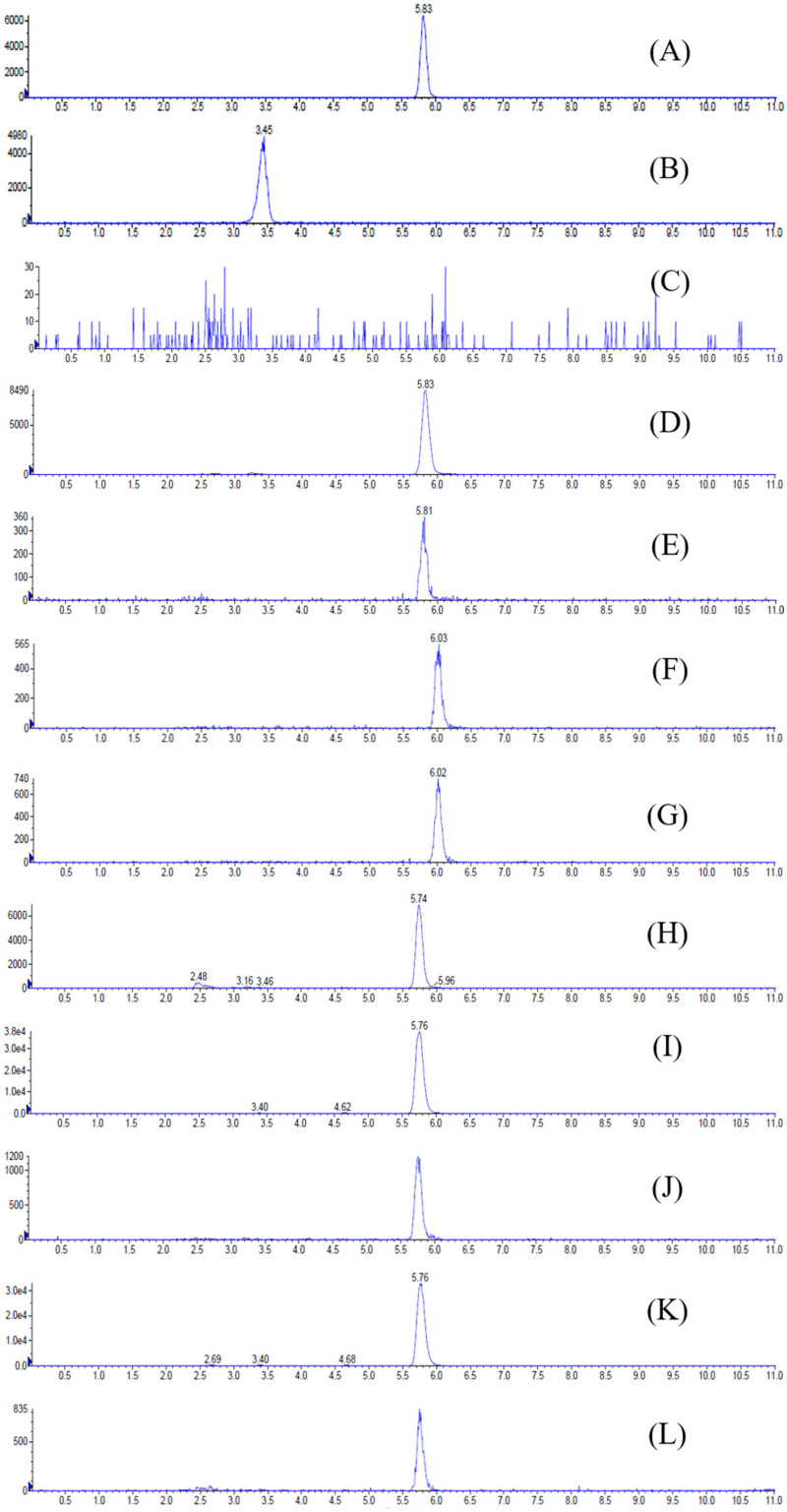
Representative HPLC Chromatograms for (**A**) erinacine A (200 ng/mL), (**B**) internal standard (2,4,5-trimethoxybenzaldehyde; 50 ng/mL), (**C**) blank rat plasma, (**D**) rat plasma sample obtained at 360 min after a 2.381 g/kg BW oral dose of *Hericium erinaceus* mycelia extract (equivalent to 50 mg/kg BW of erinacine A), (**E**) brain, (**F**) heart, (**G**) lung, (**H**) liver, (**I**) kidney, (**J**) stomach, (**K**) small intestine, and (**L**) large intestine samples obtained 4 h after a 2.381 g/kg BW oral dose of *H. erinaceus* mycelia extract.

The product ion spectrum of erinacine A and the typical chromatograms of rat plasma and tissues after administration of erinacine A are shown in Figure 1 and Figure 2, respectively. The peaks were detected with good shapes and resolutions. Retention times for erinacine A and internal standard 2,4,5-TMBA were detected at 5.83 min, and 3.45 min, respectively. No significant interference from endogenous materials was observed in the plasma or tissue samples at the retention times of erinacine A or internal standard.

The intra-day and inter-day precision and accuracy for erinacine A analyzed are shown in Table 1 and found to be within the acceptable limits (≤15%) [26]. The results of intra-day and inter-day analysis showed linear fits from 5 to 500 ng/mL with the relationship of y = 0.961x + 4.051 (R^2^ = 0.999) and y = 0.927x + 1.461 (R^2^ = 0.998), respectively. According to the results of chromatograms and analysis of precision and accuracy, the assay conditions adopted is adequate and specific to characterize the pharmacokinetics of erinacine A.

The extraction recoveries of erinacine A in rat plasma and tissues are listed in Table 2. The recoveries from plasma and tissues were higher than 75%.

### 3.2. Pharmacokinetic Parameters of Erinacine A

**Figure 3:**
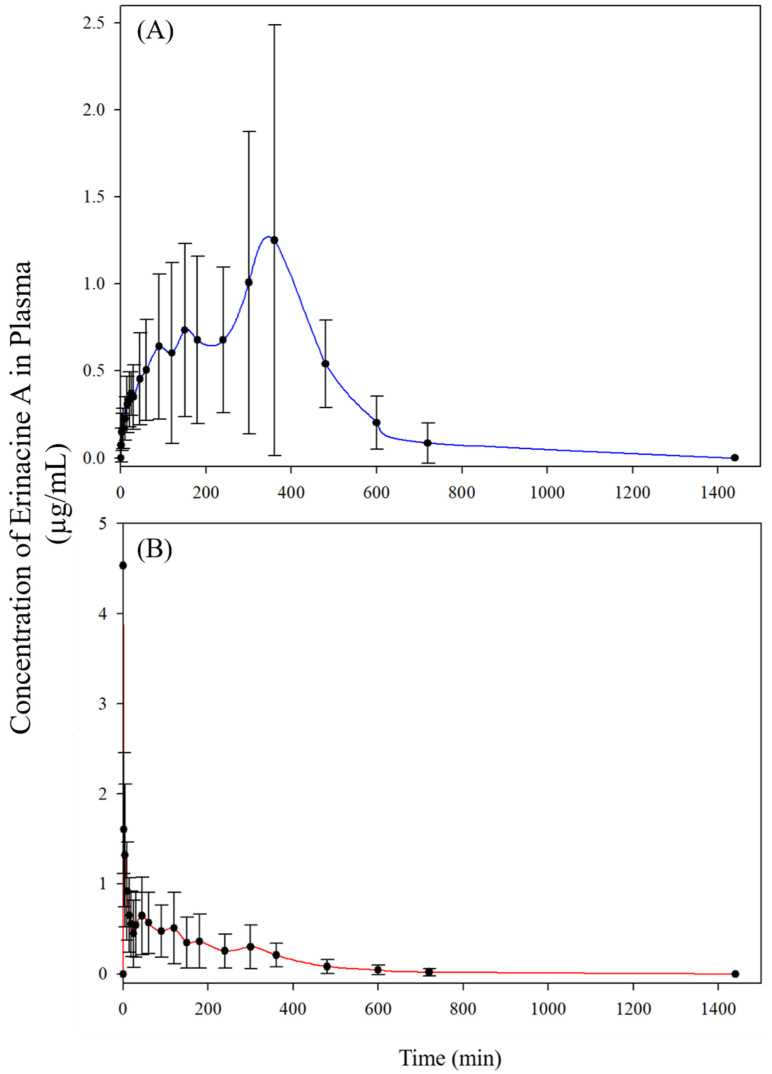
Plasma concentration–time curves of erinacine A in rats after (**A**) oral administration of *H. erinaceus* mycelia extract at 2.381 g/kg BW (equivalent to 50 mg/kg BW of erinacine A), and (**B**) intravenous administration of erinacine A at 5 mg/kg BW. Values are means ± SD (n = 6).

The time course of erinacine A after oral administration of 2.381 g/kg BW *H. erinaceus* mycelia extract (equivalent to 50 mg/kg BW of erinacine A) or intravenous injection of 5 mg/kg BW of erinacine A was monitored. The plasma concentration-time curves after oral and intravenous administrations were plotted in Figure 3 and the mean pharmacokinetic parameters were presented in Table 3. After oral administration, the maximum plasma concentration (1.40 ± 1.14 μg/mL) of erinacine A was observed at 360.00 ± 131.45 min. After reaching the peak concentration, erinacine A was gradually eliminated with the half-life of 491.22 ± 111.70 min. Right after intravenous administrations, the plasma concentration of erinacine A immediately reached the maximum of 4.53 ± 3.42 μg/mL and gradually decreased to half at 4.37 ± 4.55 min after administration. The area under the curve (AUC) values for the plasma concentration-time profiles of oral and intravenous administration were 457.26 ± 330.50 and 187.50 ± 105.29 min × μg/mL, respectively. By the equation of [(AUCpo × Doseiv)/(AUCiv × Dosepo)] × 100%, the absolute bioavailability of erinacine A in rats was estimated as 24.39%.

### 3.3. Tissue Distribution of Erinacine A

**Figure 4:**
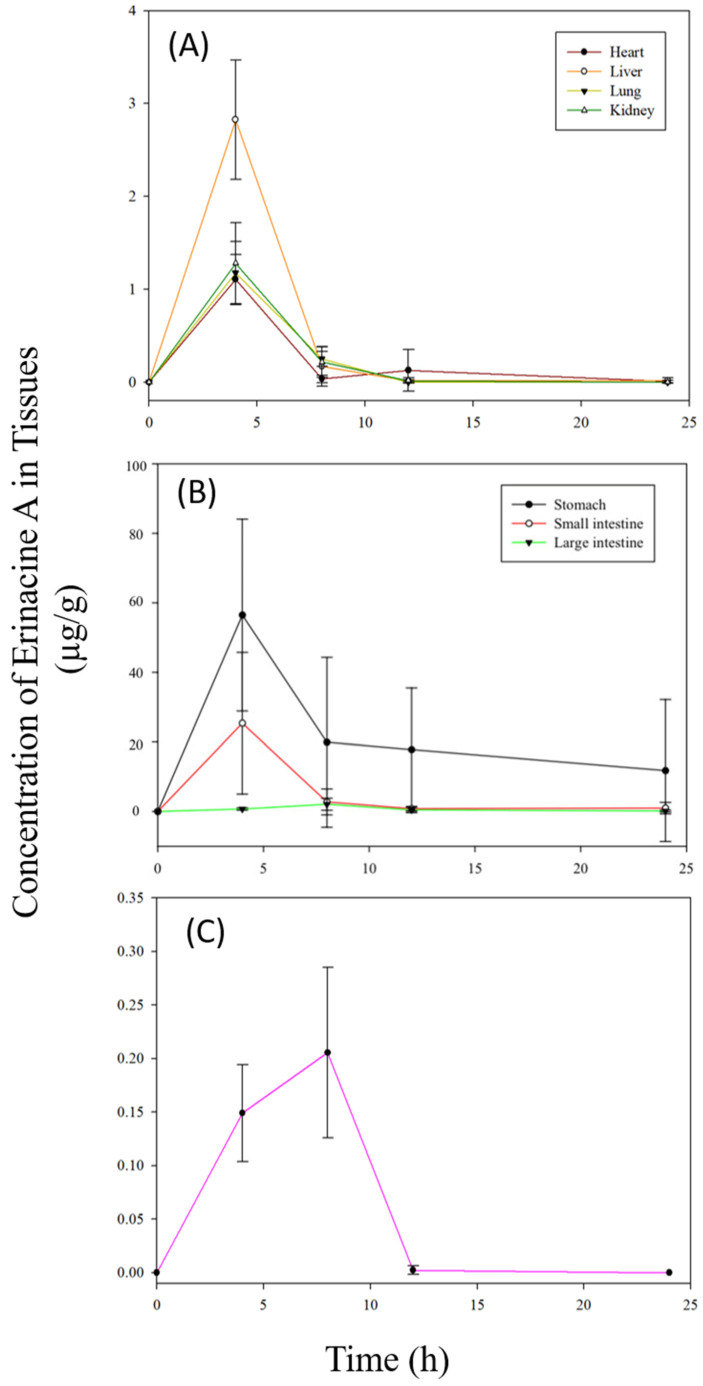
Mean plasma concentration–time curves of erinacine A in (**A**) stomach, small intestine, large intestine, (**B**) heart, liver, lung, kidney, and (**C**) brain of rats after oral administration of *H. erinaceus* mycelia extract at 2.381 g/kg BW (equivalent to 50 mg/kg BW of erinacine A). Values are means ± SD (n = 6).

After a single oral administration of *H. erinaceus* mycelia extract at 2.381 g/kg BW (equivalent to 50 mg/kg BW of erinacine A), the tissue distribution of erinacine A was determined within 24 h (Figure 4). In stomach, the concentration of erinacine A reached 56.493 ± 27.593 μg/g at 4 h after administration while the concentrations of erinacine A in small intestine, large intestine, heart, liver, lung, and kidney were detected at the same time point as 25.375 ± 20.359, 0.726 ± 0.336, 1.109 ± 0.264, 2.826 ± 0.641, 1.176 ± 0.339, 1.279 ± 0.438 μg/g, respectively. Erinacine A was detected in brain at four hours after administration and reached the maximum at eight hours (0.205 ± 0.079 μg/g). At the end of 24 h, erinacine A was still detectable in GI tract, heart, and liver.

### 3.4. Excretion of Erinacine A

Table 4 shows the fecal and urinary excretion of erinacine A within 48 h after a single oral administration of *H. erinaceus* mycelia. The fecal excretion of unchanged form of erinacine A gradually increased with time within 12 h but significantly decreased after 24 h. The maximum concentration was observed at 8–12 h (271.237 μg/g). The urinary excretion of unchanged form of erinacine A peaked at 0–4 h after oral dosing (0.248 μg/mL), but gradually decreased with time. The cumulative fecal and urinary excretion was 2.823% and 0.017% of administered dose, respectively.

### 3.5. Protein Binding of Erinacine A

**Figure 5:**
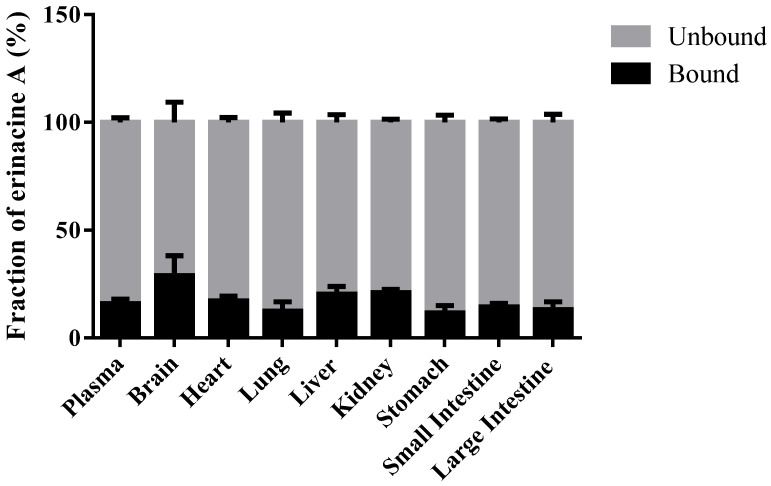
Fraction unbound and bound erinacine A in plasma and diluted (4×) tissue homogenates (brain, heart, lung, liver, kidney, stomach, large intestine, and small intestine) determined using the rapid equilibrium dialysis devices with 8000 Da molecular weight cut-off and analyzed by LC-MS/MS (n = 3). Data are presented as mean ± SD.

The preliminary study showed that the nonspecific binding of erinacine A with the plasma or tissues was found to be negligible at 37 °C up to 4 h (data not shown). Hence, after 4 h of equilibrium, the percent fractions of unbound and bound erinacine A in plasma and tissues are shown in Figure 5. The percent free erinacine A measured in plasma is 84.00 ± 2.01%, suggesting the availability for the target receptor binding and pharmacologic activity. Interestingly, the binding of erinacine A was found to be the highest as 28.94 ± 9.29% with the brain while the binding of erinacine A was found to be the lowest as 11.68 ± 3.34% with the stomach (Figure 5).

## 4. Discussion

Erinacine A-enriched *H. erinaceus* mycelia has been applied to animals, but this is the first report on pharmacokinetic studies of erinacine A following the oral administration of *H. erinaceus* mycelia extract and intravenous injection of erinacine A. Consistent with previous toxicity studies [19,20,21], no toxicity signs were observed when 2.381 g/kg of *H. erinaceus* mycelia extract or 5 mg/kg erinacine A was administered to the animals in this study.

Since supplementation of erinacine A is mostly taken as mycelia extract, freeze-dried *H. erinaceus* mycelia powder instead of pure erinacine A was chosen for oral gavage. The preliminary results showed that 1 g of freeze-dried *H. erinaceus* mycelia powder yields 21 mg of erinacine A. If the rats were to be given an equivalent of 25, 50, or 100 mg/kg body weight of erinacine A, they would have exposed to 1.190, 2.381, or 4.762 mg/kg BW freeze-dried *H. erinaceus* mycelia powder. After comparing the administration volumes and the resolutions of LC-MS/MS analysis of plasma for these three doses, 2.381 mg/kg BW mycelia powder (equivalent to 50 mg/kg BW erinacine A) was chosen for oral administration in this study.

Erinacine A was detected in plasma as early as 1 min after oral administration of mycelia powder and the concentration gradually rose up to first peak (639.79 ng/mL) at 90 min, second peak (735.19 ng/mL) at 150 min, and the third peak (1251.67 ng/mL) at 360 min. In our previous study [22], erinacine S was orally given to the rats as freeze-dried *H. erinaceus* mycelia powder at the dose of 2.395 mg/kg BW (equivalent to 50 mg/kg BW of erinacine S) and intravenously administrated as pure compound at the dose of 5 mg/kg BW. Compared to erinacine S, the time for erinacine A to reach maximum plasma concentration after oral administration was longer (360.00 vs. 270.00 min) and the maximum plasma concentration detected was higher (1.40 vs. 0.73 μg/mL). The multiple-peak phenomenon observed in the plasma profile of erinacine A after oral administration was not observed in that of erinacine S. Generally, the multiple peaking phenomena may result from different mechanisms, including chemical entity of erinacine A, delayed gastric emptying, re-entrance into digestive tract through biliary secretion, or variability of gastrointestinal absorption [28,29,30]. Previous studies have shown that low-molecular and unionized molecules are usually lipid soluble and thus can be good penetrators through membranes [31]. Furthermore, the lipophilic drug often undergoes enterohepatic circulation, which often prolongs longer half-life in a plasma concentration-time profile and is associated with the generation of one or more secondary plasma peaks [32]. In some circumstances, some very lipophilic drugs may bypass the portal circulation and gain access to the systemic circulation via the lymphatic system [33]. As erinacine S was found to be more lipophilic compared to erinacine A, the double peaks could be interpreted by the enterohepatic circulation of erinacine A, while more lipophilic erinacine S could avoid hepatic first pass metabolism by transporting through the intestinal lymphatic system. Further investigations, however, are needed to elucidate this assumption.

The fraction of the unchanged xenobiotics appeared in systemic circulation after oral administration is described as absolute bioavailability. The absolute bioavailability of erinacine A was estimated to be 24.39%, higher than 15.13% of erinacine S in the previous study [22]. The distribution of a xenobiotic in tissues is dependent on its physio-chemical properties, ability to bind plasma proteins and tissues, regional blood flow, and perfusion rate of tissues [34]. Macromolecules such as albumin and alpha-1-acid glycoprotein in blood plasma are the two major proteins in plasma that are responsible for the binding of xenobiotics in the systemic circulation [35]. In this study, the in vitro protein binding of erinacine A showed a low protein binding degree to rat plasma, which increases its availability to diffuse from the vascular system and becomes available at the tissues exert its action. One hour after oral dosing, the concentrations of erinacine A detected in stomach was not significantly different from small intestine. At 4 h, the concentration of erinacine A in stomach rose up to 56.493 ± 27.593 μg/g, but declined rapidly in small intestine as well as large intestine, liver, heart, lung, and kidney. This in-vivo result support and corroborate with the in vitro protein binding test as the tissues have a low affinity for the drug, leading to fast distribution. Interestingly, erinacine A was detected in brain 1 h after oral administration and reached the peak at 8 h. Combing the protein binding assay and using the previously reported equation to calculate the unbound brain to plasma concentration ratio [36], the unbound erinacine A brain-to-plasma concentration ratio is close to unity, supporting that erinacine A penetrate the blood-brain barrier via passive diffusion as the influx and efflux clearance rates are similar. Our previous study showed that erinacine S can penetrate blood-brain barrier [22]. However, this study not only is the first to show that erinacine A is able to cross blood-brain barrier but also by the means of passive diffusion. Although the amount of unchanged erinacine A detected in brain was less than that of unchanged erinacine S, in-vivo studies have shown the neuroprotective properties of erinacine A [8,9,10,11,13,18,37]. The biotransformation products of erinacine A formed in GI tract, circulation, or tissues would contribute to its neurohealth effects.

The amounts of unchanged erinacine A measured in tissues at 24 h period after oral dosing were less than those of unchanged erinacine S measured in previous study [22]. This could be explained by the observation that the total amounts of unchanged erinacine A collected in feces and urine for 24 h were more than 20 and 2 times, respectively, higher than those of erinacine S collected. The unchanged compound appeared in feces reflexes the amount of the compound not absorbed through digestive epithelium and not metabolized by enzymes or microorganisms in GI tract. The unchanged compound appeared in urine implies the amount that reached the systemic circulation but not transported into tissues.

## 5. Conclusions

In this study, a fully validated LC-MS/MS method was applied to investigate the pharmacokinetic disposition of erinacine A, including its bioavailability in rats following IV and PO administration, its tissue distribution, and its protein binding properties. Results showed that the absolute bioavailability of erinacine A in Sprague-Dawley rats after oral dosing at 2.381 g/kg BW *H. erinaceus* mycelia extract (equivalent to 50 mg/kg BW of erinacine A) was 24.39%. The concentration of erinacine A reached the peak in the stomach, small intestine, large intestine, liver, heart, lung, and kidney at 4 h and reached the peak in the brain at 8 h. This in-vivo result supports and corroborates the in-vitro protein binding test as various tissues have a low affinity for the drug, except for the brain. Moreover, this study is the first to show that erinacine A can penetrate the blood-brain barrier of rats by the means of passive diffusion and support the development of *H. erinaceus* mycelia for the improvement of neurohealth. However, for drug development, metabolomic profiles of erinacine A and its oral availability in humans warrant further studies.

## Figures and Tables

**Table 1:** Intra-day and inter-day precision and accuracy of LC/MS/MS method for the determination of erinacine A.

	Intra-Day			Inter-Day		
Theoretical Conc.	Observed Conc.	Precision	Accuracy	Observed Conc.	Precision	Accuracy
(ng/mL)	(ng/mL)	(% CV)	(% bias)	(ng/mL)	(% CV)	(% bias)
5	4.27 ± 0.37	8.79	−14.70	4.08 ± 0.52	9.34	−9.66
10	12.85 ± 0.68	8.23	14.00	11.40 ± 0.94	4.04	10.33
20	17.48 ± 0.61	3.50	−12.58	16.99 ± 0.98	5.76	−15.00
50	52.55 ± 1.04	1.98	5.10	49.69 ± 1.28	2.59	−0.62
100	104.50 ± 1.76	1.68	4.50	103.49 ± 12.03	11.63	3.49
200	207.83 ± 2.40	1.16	3.92	178.67 ± 9.19	5.14	−10.67
500	479.17 ± 8.84	1.85	−4.17	466.33 ± 31.11	6.67	−6.73

Data expressed as means ± SD (n = 6). CV (%) = (standard deviation/mean) × 100%. bias (%) = [(measured concentration − theoretical concentration)/theoretical concentration] × 100%.

**Table 2:** Extract recoveries (%) of erinacine A in rat plasma and tissues.

	Theoretical Concentration (ng/mL)		
	50	200	500
Plasma	78.23 ± 9.56	94.48 ± 2.06	79.57 ± 1.99
Brain	75.85 ± 3.10	99.46 ± 2.01	86.11 ± 4.76
Heart	80.68 ± 3.99	98.43 ± 1.52	94.47 ± 2.79
Liver	88.93 ± 4.48	98.65 ± 1.23	94.42 ± 7.18
Lung	93.11 ± 17.63	94.20 ±4.24	87.32 ± 3.01
Kidney	84.61± 1.86	98.28 ± 1.19	82.24 ± 2.09
Stomach	81.01 ± 10.44	80.95 ± 7.41	98.47 ± 3.83
Small Intestine	85.52 ± 6.25	99.42 ± 3.58	95.75 ± 5.50
Large Intestine	87.63 ± 5.05	96.32 ± 1.85	99.99 ± 1.31
Feces	92.08 ± 5.22	98.71 ± 10.74	100.07 ± 2.95
Urine	75.55 ± 4.14	96.06 ± 2.35	89.14 ± 4.55

Data expressed as means ± SD (n = 6). Recovery (%) = (measured concentration / theoretical concentration) × 100%.

**Table 3:** Pharmacokinetic parameters of erinacine A in rat plasma after oral administration of *H. erinaceus* mycelia extract at 2.381 g/kg body weight (equivalent to 50 mg/kg BW of erinacine A) and intravenous administration of erinacine A at 5 mg/kg.

	P.O.	I.V.
	(50 mg/kg)	(5 mg/kg)
T_max_ (min)	360.00 ± 131.45	−
C_max_ (μg/mL)	1.40 ± 1.14	4.53 ± 3.42
T_1/2_ (min)	491.22 ± 111.70	4.37 ± 4.55
AUC (min × μg/mL)	457.26 ± 330.50	187.50 ± 105.29
Absolute Bioavailability (%)	24.39	

P.O.: oral administration; iv: intravenous administration. Data expressed as mean ± SD (n = 6). T_max_: the time taken to reach the maximum concentration. C_max_: maximum plasma concentration. T_1/2_: half-life. AUC: area under the plasma concentration-time curve. Absolute bioavailability (%) = [(AUCpo × Doseiv)/(AUCiv × Dosepo)] × 100%.

**Table 4:** Fecal and urinary excretion of erinacine A after oral administration of *H. erinaceus* mycelia extract at 2.381 g/kg BW (equivalent to 50 mg/kg BW of erinacine A).

Time (h)	Feces Concentration (μg/g)	Urine Concentration (μg/mL)
0–4	1.533 ± 2.625	0.248 ± 0.210
4–8	81.853 ± 99.919	0.118 ± 0.103
8–12	271.237 ± 404.357	0.059 ± 0.043
12–24	122.824 ± 198.611	0.055 ± 0.066
24–36	4.475 ± 6.477	0.021 ± 0.037
36–48	0.062 ± 0.099	0.016 ± 0.018
Total Amount (μg) (% of administered dose)	428.379 ± 368.502 (2.823%)	2.604 ± 2.149 (0.017%)

Data expressed as means ± SD (n = 6).

## Data Availability

Data sharing not applicable.

## Sample Availability

Samples of the compounds are not available from the authors.
